# Supplementary material for: WAVE2 Regulates Actin-Dependent Processes Induced by the B Cell Antigen Receptor and Integrins
Source: Cells. 2023 Nov 25;12(23):2704. doi: 10.3390/cells12232704 (PMC10705906; doi:10.3390/cells12232704)
Supplement: Supplementary file 1 [file cells-12-02704-s001.zip › cells-2699895-supplementary.pdf]

cells MDPI

Supplementary material

WAVE2 Regulates Actin-Dependent Processes Induced by the B Cell Antigen Receptor and Integrins

Abhishek Bedi [1], Kate Choi [1], Connor Keane [1], Madison Bolger-Munro [1], Ashley Ambrose [1] and Michael R. Gold [1,*]

[1]Department of Microbiology and Immunology and the Life Sciences Institute, University of British Columbia, Vancouver, BC, Canada V6T1Z3

*Correspondence: mgold@mail.ubc.ca (M.R.G.)

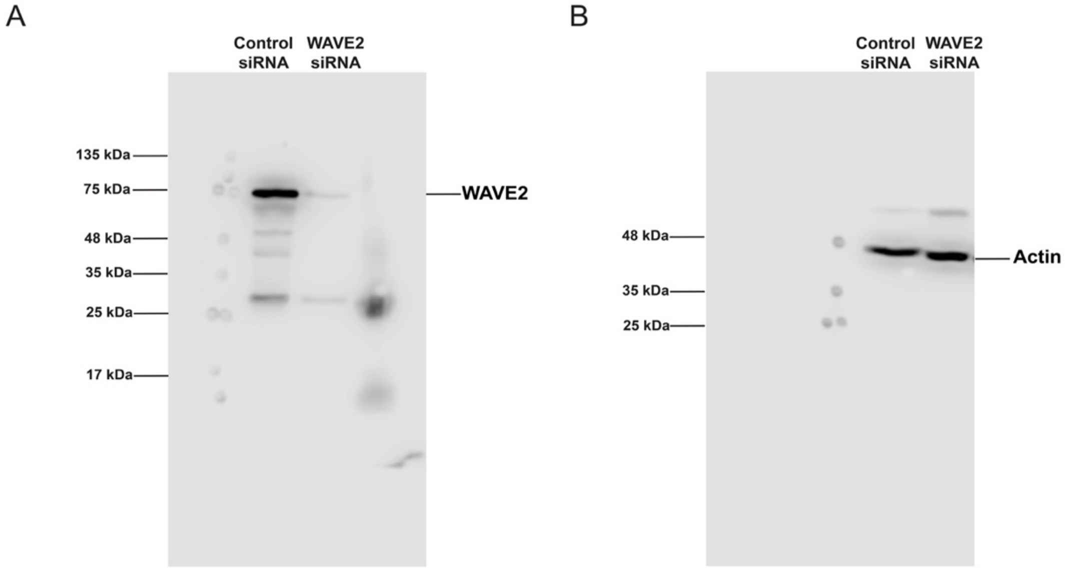

Figure S1A,B. Uncropped images of blots shown in Figure 1A (left panel). A20 cells that had been transfected with control siRNA or WAVE2 siRNA were analyzed by immunoblotting with antibodies to WAVE2 (A) or actin (B; loading control). Molecular weight standards, in kilodaltons (kDa), are indicated to the left of each blot.

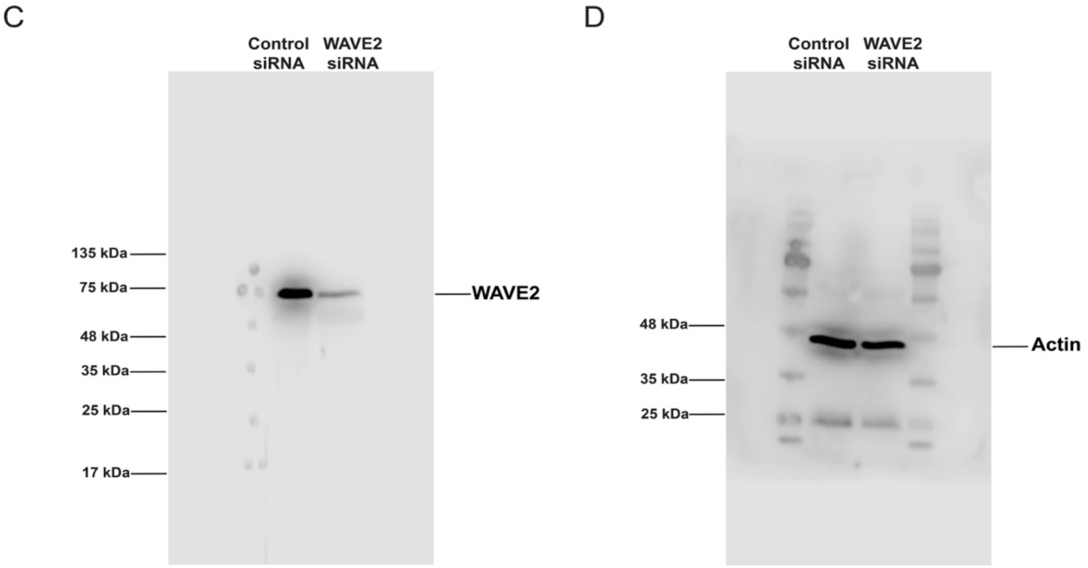

Figure S1C,D. Uncropped images of blots shown in Figure 1A (right panel). Primary B cells that had been transfected with control siRNA or WAVE2 siRNA were analyzed by immunoblotting with antibodies to WAVE2 (C) or actin (D; loading control).

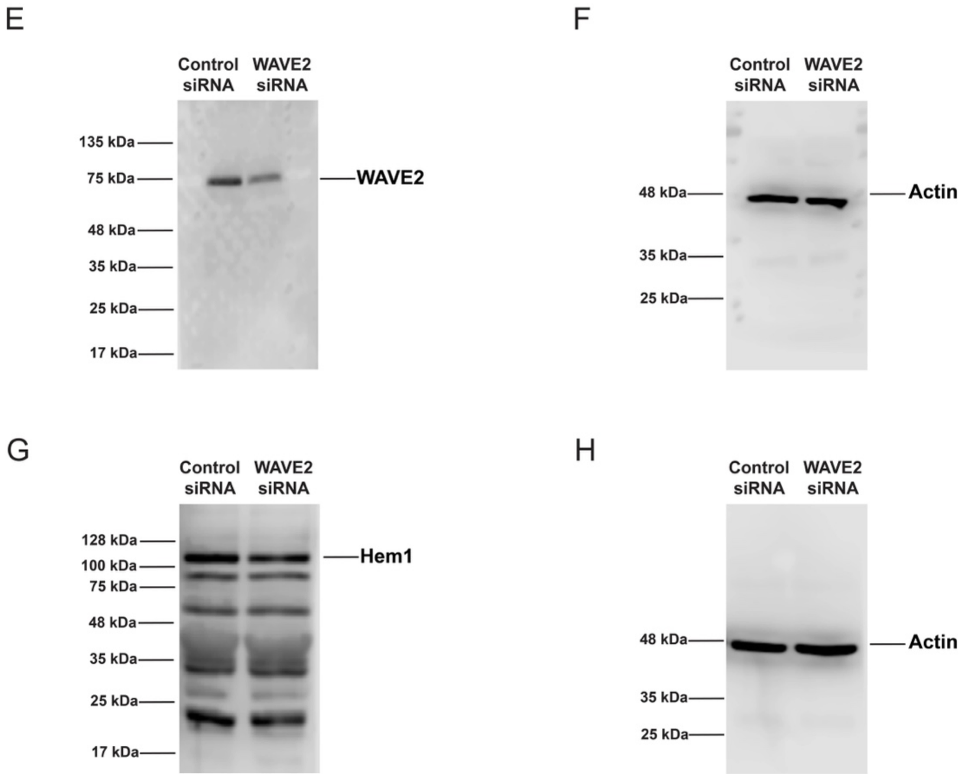

Figure S1E-H. Uncropped images of blots shown in Figure 2A. A20 cells that had been transfected with control siRNA or WAVE2 siRNA were analyzed by immunoblotting with antibodies to WAVE2 (E) or actin (F; loading control) or with antibodies to Hem1 (G) or actin (H; loading control). A similar Hem1 blot with multiple lower molecular weight bands was obtained by Salzer *et al.* (Sci. Immunol., vol. 5, eabc3979, 2020, doi: 10.1126/SCIIMMUNOL.ABC3979; see the full uncropped blot for Figure 1C in the supplementary data file for this paper).

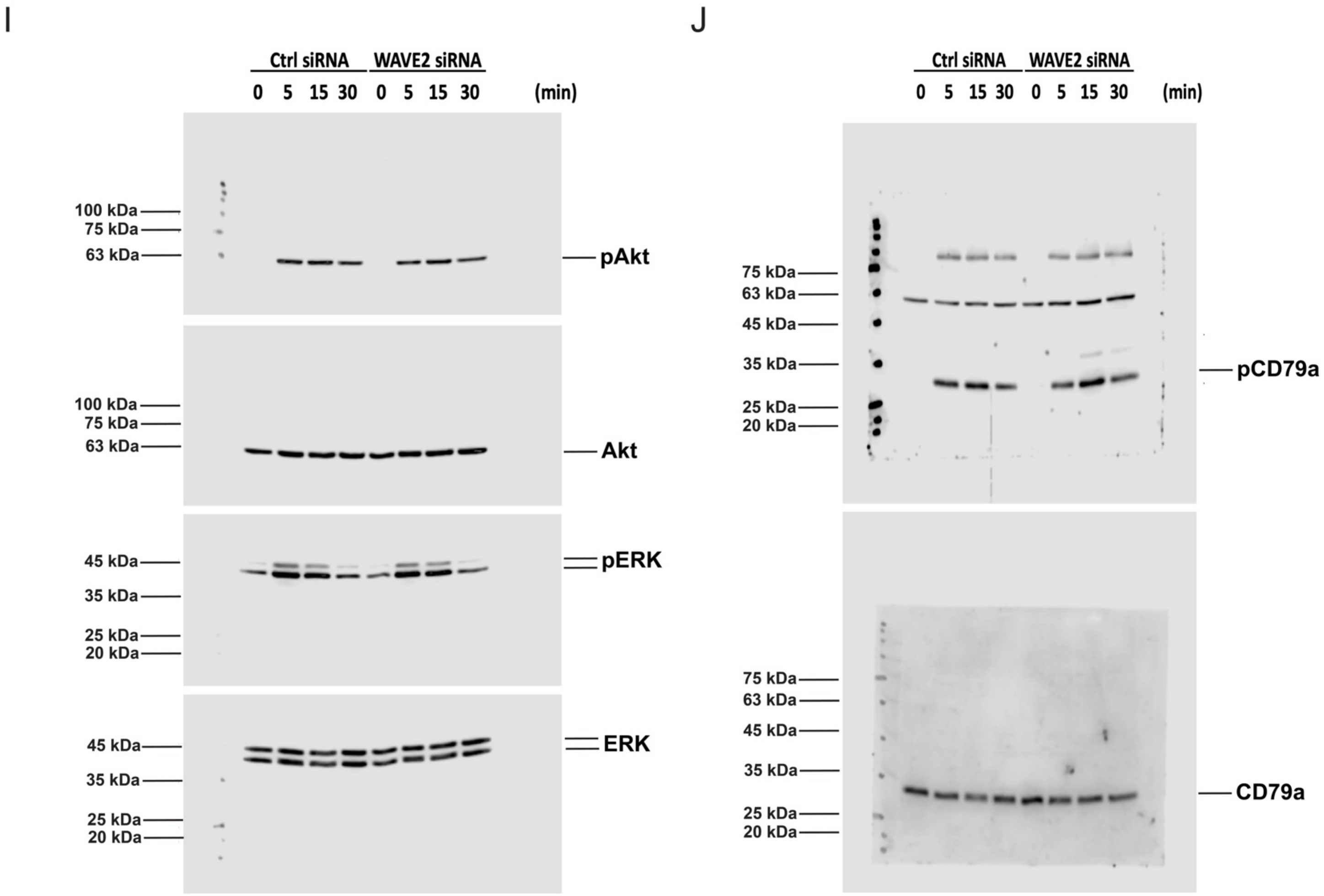

Figure S1I,J. Uncropped images of blots shown in Figure 2B. A20 cells that had been transfected with control siRNA or WAVE2 siRNA were allowed to spread on anti-IgG-coated tissue culture wells for the indicated times before analyzing BCR signaling by immunoblotting with antibodies to (I) phosphorylated Akt (pAkt), total Akt, phosphorylated ERK (pERK), and total ERK or (J) phosphorylated CD79a (pCD79a) and total CD79a.

cells

MDPI

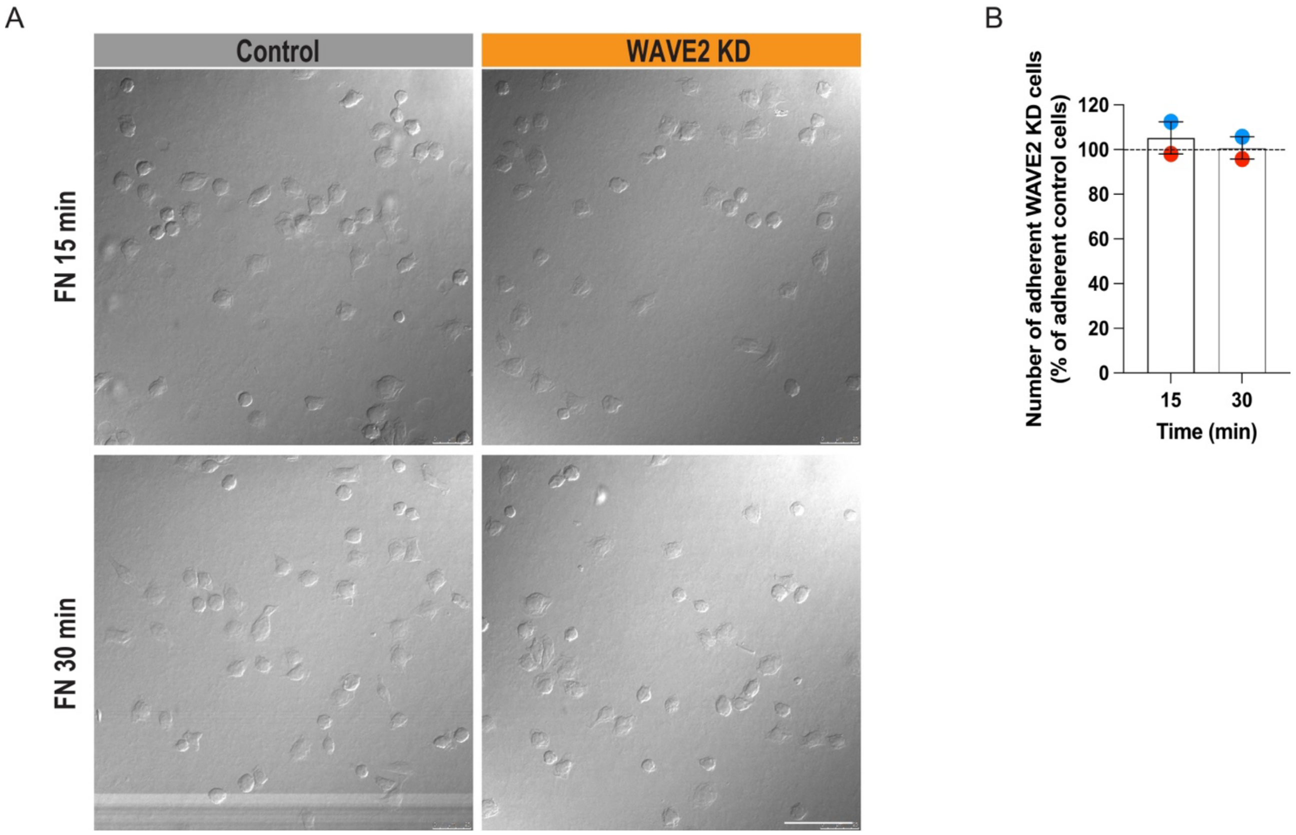

Figure S2. WAVE2 KD does not affect A20 cell adhesion to FN. A20 cells that had been transfected with control siRNA or WAVE2 siRNA were allowed to spread on FN-coated coverslips for 15 or 30 min. After changing the medium to remove non-adherent cells, firmly attached cells were imaged by spinning disk confocal microscope. (A) Images of representative fields of view. Scale bar: 10 µm. (B) The number of cells in 10 random fields of view was determined each condition and the total number of adherent WAVE2 KD cells at each time point is expressed as a percent of the values for the control cells. Each dot is an independent experiment. The data are presented as the average ± range for two experiments.
